# Cell-Type-Dependent Role for nsP3 Macrodomain ADP-Ribose Binding and Hydrolase Activity during Chikungunya Virus Infection

**DOI:** 10.3390/v14122744

**Published:** 2022-12-09

**Authors:** Taewoo Kim, Rachy Abraham, Lisa Pieterse, Jane X. Yeh, Diane E. Griffin

**Affiliations:** W. Harry Feinstone Department of Molecular Microbiology and Immunology, Johns Hopkins Bloomberg School of Public Health, Baltimore, MD 21205, USA

**Keywords:** chikungunya virus, macrodomain, alphavirus, ADP ribosylation, replication complexes, viral replication, host-virus interaction, astrocytes, neurons, neurovirulence

## Abstract

Chikungunya virus (CHIKV) causes outbreaks of rash, arthritis, and fever associated with neurologic complications, where astrocytes are preferentially infected. A determinant of virulence is the macrodomain (MD) of nonstructural protein 3 (nsP3), which binds and removes ADP-ribose (ADPr) from ADP-ribosylated substrates and regulates stress-granule disruption. We compared the replication of CHIKV 181/25 (WT) and MD mutants with decreased ADPr binding and hydrolase (G32S) or increased ADPr binding and decreased hydrolase (Y114A) activities in C8-D1A astrocytic cells and NSC-34 neuronal cells. WT CHIKV replication was initiated more rapidly with earlier nsP synthesis in C8-D1A than in NSC-34 cells. G32S established infection, amplified replication complexes, and induced host-protein synthesis shut-off less efficiently than WT and produced less infectious virus, while Y114A replication was close to WT. However, G32S mutation effects on structural protein synthesis were cell-type-dependent. In NSC-34 cells, E2 synthesis was decreased compared to WT, while in C8-D1A cells synthesis was increased. Excess E2 produced by G32S-infected C8-D1A cells was assembled into virus particles that were less infectious than those from WT or Y114A-infected cells. Because nsP3 recruits ADP-ribosylated RNA-binding proteins in stress granules away from translation-initiation factors into nsP3 granules where the MD hydrolase can remove ADPr, we postulate that suboptimal translation-factor release decreased structural protein synthesis in NSC-34 cells while failure to de-ADP-ribosylate regulatory RNA-binding proteins increased synthesis in C8-D1A cells.

## 1. Introduction

Chikungunya virus (CHIKV) is an enveloped, positive-sense RNA alphavirus in the *Togaviridae* family that is transmitted mainly by *Aedes* spp. mosquitoes. CHIKV predominantly causes arthritis and rash accompanied by fever [1]. However, recent outbreaks of CHIKV infection have included a variety of neurologic complications including encephalomyelitis, Guillain-Barre syndrome, neonatal hypotonia, and neuro-ocular disease [2]. Studies of CHIKV neurotropism in mice and macaques have shown preferential infection of astrocytes rather than neurons [3,4] and in vitro studies confirm efficient replication and cytopathic effects in astrocytes and glial cells [5,6,7]. Astrocyte tropism was also observed during in vivo stereotaxic delivery of a CHIKV-pseudotyped vector to adult rat brain [8]. Astrocytes are the most abundant cells in the central nervous system (CNS) and important for regulating the microenvironment through development and maintenance of the blood–brain barrier, metabolic support for neurons, synaptic transmission, and inflammatory and immune responses as sensors of infection [9,10]. However, most arboviruses that cause encephalitis replicate preferentially in neurons [11,12,13,14,15] and the basis for astrocyte-associated CHIKV neurovirulence is not known.

The CHIKV genome is a single-stranded 11.8 kb RNA with a cap at the 5′terminus and a poly (A) tail at the 3′terminus [16,17]. The genome is organized in two open reading frames (ORFs): the 5′ ORF encodes the four nonstructural replicase proteins (nsPs) and the 3′ ORF encodes the structural proteins for virion assembly: capsid and envelope glycoproteins E1 and pE2. As a first step in replication after genome delivery, the nsPs are translated from the genomic RNA (gRNA) as polyproteins P123 and P1234, which are systematically processed by the nsP2 protease [18]. Except for nsP3, the functions of nsPs in replication are well-understood [19,20,21,22,23]. nsP1 has capping activities, anchors the replication complex to membranes, and initiates minus-strand synthesis [24,25,26,27,28]; nsP2 has helicase, NTPase, RNA triphosphatase, and protease activities [29,30,31,32,33]; and nsP4 is the RNA-directed RNA polymerase [34]. 

nsP3 is a determinant of neurovirulence that functions as part of the polyproteins P123 and P23 and as an individual protein [35,36,37,38,39]. With the translation of incoming gRNA and initiation of infection, spherules that contain replication complexes are formed at the plasma membrane where sequential processing of the nonstructural polyprotein regulates the synthesis of viral negative-strand RNA as well as genomic and subgenomic (sg) positive-strand RNAs [17]. nsP3 is present both in these membrane-bound complexes and later in large cytoplasmic complexes (nsP3 granules). Infection induces RNA-dependent protein kinase (PKR)-mediated phosphorylation of eIF2α, cessation of host protein and viral nsP synthesis and ADP-ribosylation-dependent aggregation of translation initiation factors (eIFs) and RNA-binding proteins (RBPs) from stalled ribosomes into stress granules that are disrupted by the formation of nsP3 granules [40,41]. nsP3 disrupts or prevents the formation of stress granules by binding RBPs, including essential stress granule protein Ras GTPase-activating (SH3 domain) protein-binding protein (G3BP1), to form distinct nsP3 granules and release eIFs [42,43,44,45]. 

nsP3 has three major domains: a highly phosphorylated, poorly conserved C-terminal hypervariable domain (HVD), a zinc-binding alphavirus unique domain, and a highly conserved N-terminal macrodomain (MD). The HVD is a hub for interactions with cellular proteins required for replication-complex formation and the initiation of RNA synthesis [46,47,48]. MDs are present in togaviruses, coronaviruses, and hepatitis E virus, and are determinants of viral virulence and intolerant of mutation, but why they are so essential for replication has not been determined. Viral MDs share the ability to bind ADPr and to remove this post translational modification from ADP-ribosylated proteins and are multifunctional with features that are likely cell-type and virus-strain dependent [38,49,50].

ADP ribosylation is catalyzed by a family of ADP-ribosyltransferases (ARTs; commonly called poly ADP ribosyl polymerases or PARPs), which transfer ADPr from NAD^+^ onto target proteins [51,52]. The transfer of ADPr moieties regulates protein function and occurs either singly as mono ADPr (MAR) or in multiple units through linear or branched polymeric chains as poly ADPr (PAR). ADP ribosylation is induced during CHIKV infection of neuronal cells and the inhibition of ART activity reduces viral replication, suggesting that interaction with an ADP-ribosylated host or viral protein is important for replication [53]. Alphaviruses with MD mutations that severely impair binding and/or hydrolase activity are not viable due to a failure to synthesize RNA, indicating an essential function for this domain [37,53]. However, viruses with mutations that decrease ADPr-binding and/or reduce ADP ribosylhydrolase activity can be recovered for functional studies. These mutants have impaired replication, particularly in differentiated cells, and decreased virulence in mice [37,38,49,54,55].

Previous studies of the alphavirus MD have demonstrated its role in neurovirulence and viral replication in neuronal cells [37,38,53,55], while studies of the coronavirus MD have focused primarily on the modulation of host immune responses and replication in astrocytes [56,57]. In the CHIKV MD, glycine at position 32 binds the adenine of ADPr and modulates hydrolase activity while the NH group of tyrosine at 114 binds the phosphate and coordinates the distal ribose of ADPr [38,58,59]. Studies of CHIKV with MD mutations G32S (decreased ADPr binding and hydrolase activities) and Y114A (increased ADPr binding but decreased hydrolase activity) in NSC-34 mouse neuronal cells showed that ADPr binding is necessary for the initiation of virus replication and translation of nsPs and RNA synthesis, while hydrolase activity facilitates amplification of replication complexes [53]. Studies in human U2OS cells have shown that nsP3 MD hydrolase activity is required for stress-granule suppression, release of eIFs, and the removal of ADPr from RBPs [42]. However, MD function in CHIKV-infected astrocytes has not been investigated.

To examine the cell-type dependence of CHIKV replication, we have compared the infection of C8-D1A astrocytic cells to previously studied NSC-34 neuronal cells. Infection with WT CHIKV was initiated more efficiently in C8-D1A cells than in NSC-34 cells, but MD mutations had similar effects on the early stages of infection. However, there were substantively different effects on the translation of sgRNA to produce the structural proteins. In NSC-34 cells, ADPr binding and hydrolase deficiency decreased the synthesis of structural proteins compared to WT, while in C8-D1A cells synthesis was increased, demonstrating the importance of cell-type in determining MD function for alphavirus replication.

## 2. Materials and Methods

### 2.1. Cell Culture

The murine astrocyte type I clone (C8-D1A, CRL-2541; [60]), baby hamster kidney 21 (BHK 21), and African green monkey epithelial (Vero) cells were obtained from the American Type Culture Collection (Manassas, VA, USA). Murine neuronal NSC-34 cells were a gift from Neil Cashman, University of British Columbia, Canada [61]. All cell lines were documented to be free of mycoplasma (Lonza Bioscience, Walkersville, MD, USA) and cultured at 37 °C and 5% CO_2_ in Dulbecco’s modified Eagle’s medium (DMEM; Gibco, Grand Island, NY, USA) supplemented with 10% heat-inactivated fetal bovine serum (FBS; Atlanta Biologicals, Flower Branch, GA, USA), streptomycin (100 μg/mL; Gibco, Grand Island, NY, USA), penicillin (100 U/mL; Gibco, Grand Island, NY, USA), and L-glutamine (2 mM; Gibco, Grand Island, NY, USA). 

### 2.2. Viruses, Infections, and Assays

Viral RNA was transcribed from a full-length cDNA clone of CHIKV vaccine strain 181/25 (a gift from Naomi Forrester, University of Texas Medical Branch at Galveston, TX) using a mMESSAGE mMACHINE SP6 Kit (Invitrogen, Waltham, MA, USA) [62,63]. Point mutations were introduced into the 181/25 cDNA clone in the nsP3 MD to create two viable nsP3 MD mutant viruses, G32S and Y114A, and rescued in BHK-21 cells as previously described [38]. Recombinant viruses were passaged in BHK-21 cells, and viral RNAs were sequenced to confirm the mutations. Virus titers were determined by plaque assay in Vero cells. Virus replication in NSC-34 and C8-D1A cells was assessed after infection at an MOI of 5 based on plaque formation in Vero cells. Data are expressed as PFU/mL. Cell viability was determined by trypan blue exclusion and expressed as a percentage of day 0 cells. 

### 2.3. Infectious Center Assays

C8-D1A cells were infected with WT or nsP3 MD mutants at MOIs of 0.5 and 5 and incubated for 1h at 4 °C and then at 37 °C for 4 h, trypsinized, counted, serially diluted in DMEM supplemented with 1% heat-inactivated FBS and plated on Vero cells. To bypass the entry step, C8-D1A cells were electroporated with 10 µg of RNA from a full-length clone of CHIKV 181/25 (WT) or nsP3 MD mutants using the Amaxa nucleofector [53]. Transfected cells were trypsinized, serially diluted, and plated on 90% confluent BHK-21 cells. Co-cultured cells were overlaid with 0.6% bacto agar in MEM, incubated at 37 °C for 48 h, then fixed and stained with crystal violet for the identification of plaques. Data are expressed as infectious centers per 10^5^ cells/mL.

### 2.4. Flow Cytometry for dsRNA

C8-D1A cells were infected at an MOI of 5 or mock-infected and incubated at 37 °C. At indicated times after infection, the cells were trypsinized with live/dead staining (Invitrogen, Waltham, MA, USA) for 30 min on ice in the dark. Cells were then fixed with 2% formaldehyde in PBS for 30 min and permeabilized with 0.2% Triton in FACS buffer (PBS with 0.4% 0.5 mM EDTA and 0.5% BSA) for 30 min on ice. Cells were stained for dsRNA with J2 mouse monoclonal antibody (1:1000; Scicons, Szirak, Hungary) for one hour followed by secondary PE-conjugated anti-mouse IgG antibody (1:400) for 45 min [53]. Cells were analyzed on a FACS Canto flow cytometer (Becton-Dickinson, San Jose, CA, USA). Histograms were plotted, and the percent of live cells positive for dsRNA and median fluorescence intensity were quantitated.

### 2.5. qRT-PCR for Viral RNA

RNeasy Plus mini kit (Qiagen, Germantown, MD, USA) was used to isolate total RNA from cells infected at an MOI of 5. cDNA was synthesized from RNA using the High Capacity cDNA reverse transcription kit (Applied Biosystems, Waltham, MA, USA). qRT-PCR was performed using TaqMan primers and probes specific for the E2 region of the CHIKV genome and subgenome: E2 922F 5′-GAAGAGTGGGTGACGCATAAG-3′; E2 1011R 5′-TGGATAACTGCGGCCAATAC-3′); TaqMan probe: CHK E2 949 5′-6-carboxyfluorescein [FAM]-ATCAGGTTAACCGTGCCGACTGAA-Minor groove binder (MGB) nonfluorescent quencher (NFQ)-3′ (Applied Biosystems, Waltham, MA, USA). Copies of viral RNA were quantified using a standard curve constructed from 10-fold dilutions of a pCRII-TOPO plasmid containing the CHIKV E2 region cDNA and normalized to endogenous rodent *Gapdh*. Data are expressed as mean CHIKV RNA copies per 10^6^ copies of *Gapdh* [53].

### 2.6. Immunoblot Analyses of Viral Protein Expression

Cells were infected at an MOI of 5 or mock-infected and incubated at 37 °C for different times. At each time point, cells were lysed in radioimmunoprecipitation assay (RIPA) buffer (50 mM Tris (pH 8), 1% Triton X-100, 0.1% SDS, 150 mM NaCl, 1 mM EDTA, and 0.5% Na_3_VO_4_2H_2_O) supplemented with protease (Sigma-Aldrich, St. Louis, MO, USA) and phosphatase (Roche, Basel, Switzerland) inhibitors. Lysates were incubated on ice for 30 min followed by centrifugation at 15,200× *g* for 10 min. The DC protein assay (Bio-Rad, Hercules, CA, USA) was used to estimate total protein using BSA as the standard. A total of 15 µg of proteins was loaded onto 10% polyacrylamide gels, separated by electrophoresis, and transferred to nitrocellulose membranes. Membranes were blocked with 5% skim milk in PBS and incubated overnight at 4 °C with polyclonal rabbit antibodies to CHIKV nsP1, nsP2, nsP3 (1:10000, a gift from Prof. Andres Merits, University of Tartu, Estonia), mouse monoclonal antibodies to CHIKV E2 (1:1000, CHK-187 11A4. F1.F4, a gift from Prof. Michael Diamond, Washington University, St. Louis, MO, USA), phospho-eIF2α, eIF2α (1:1000; Cell Signaling Technologies, Danvers, MA, USA), or β actin (1:5000; MilliporeSigma, Burlington, MA, USA) diluted in 5% BSA in tris-buffered saline and Tween 20 (TBST). Secondary antibodies were HRP-conjugated either anti-mouse or anti-rabbit IgG (1:1000; Cell Signaling Technologies, Danvers, MA, USA) diluted in 2% milk and incubated for one hour at RT. ECL Prime Western Blotting Detection Reagent (GE Healthcare, Chicago, IL, USA) was used to develop the membranes. ImageJ software from NIH was used to analyze the densitometry of immunoblots from three to four independent experiments.

### 2.7. Pulse-chase Analysis of PE2 Protein Processing

Cells were infected and incubated at 37 °C for indicated times, then washed with PBS and incubated in DMEM without methionine (Met) and cysteine (Cys) (Gibco, Grand Island, NY, USA) for one hour. The cells were then pulsed with Met/Cys-free DMEM containing ^35^S (100 µCi/mL, PerkinElmer, Waltham, MA, USA) for 30 min at 37 °C and 5% CO_2_. To chase the labeled proteins, cells were washed with PBS and incubated with media containing 15 mg/L L-Met. At the indicated times, supernatant fluids were collected and the cells lysed in RIPA buffer containing phosphatase and protease inhibitors.

For immunoprecipitation, the DC assay was used to estimate the total protein in cell lysates. A total of 300 to 400 µg of protein was incubated with 10 µg of mouse anti-E2 mAb at 4 °C overnight with gentle rocking. A 40 µL quantity of prewashed Pierce ImmunoPure Immobilized Protein A/G (Thermo Fisher Scientific, Waltham, MA, USA) beads were added to the mixture of cell lysate and E2 antibody (CHK-187) followed by overnight incubation at 4 °C with gentle rocking. Beads were washed four times with RIPA buffer, and bound proteins were eluted by boiling with SDS loading buffer. For supernatant fluids, 1/4th volume of polyethylene glycol solution (PEG 8000 and NaCl, pH 7.2) was added, incubated overnight at 4 °C, and centrifuged at 13,500× *g* for 30 min to pellet PEG-precipitated virions. The pellet was boiled in SDS loading buffer. Eluted lysates and PEG-precipitated virions from supernatant samples were loaded onto 10% polyacrylamide gels and electrophoresed. Gels were fixed with fixing solution (10% acetic acid and 20% methanol) for 30 min and washed with water for an hour with water replacement every 15 min. Gels were enhanced with 1M sodium salicylate (Sigma-Aldrich, St. Louis, MO, USA) solution (pH 5 to 7) for 30 min, dried at 56 °C, and exposed to ECL X-ray (GE Healthcare, Chicago, IL, USA) film.

### 2.8. Extracellular Genome: PFU Ratio

Cells infected at an MOI of 5 for an hour were washed thrice with PBS to remove the unadsorbed virus, and fresh DMEM + 1% FBS was added. At indicated times, supernatant fluids were collected and centrifuged to remove any debris. Infectious virus was quantified by plaque assay and RNA was isolated using the QIAamp viral RNA kit (Qiagen, Venlo, The Netherlands). qRT-PCR was performed using TaqMan nsP2 primers and probes specific for the CHIKV genome: nsP2 1247F 5′-GTACGGAAGGTAAACTGGTATGG-3′; nsP2 1359R 5′-TCCACCTCCCACTCCTTAAT-3′); and TaqMan probe: CHIKV nsP2 1304 5′-(FAM)-TGCAGAACCCACCGAAAGGAAACT-(MGB NFQ)-3′ (Applied Biosystems). Copies of viral RNA were quantified using a standard curve constructed from 10-fold dilutions of RNA in vitro transcribed from pCRII-TOPO plasmid containing the CHIKV nsP2 cDNA. Genomic copies: PFU ratio was calculated at the indicated time points by converting qRT-PCR genomic copy per reaction data to genomic copies per mL. Genomic copies/mL were then divided by PFU/mL at each time point to obtain the genomic-copies-to-PFU ratio.

### 2.9. Puromycin Translation Assay

Cells were infected or mock-infected and incubated at 37 °C. After an hour of infection, fresh DMEM with 1% FBS was substituted. At indicated time points, the media were removed, and DMEM containing 5 µg/mL puromycin dihydrochloride (Sigma-Aldrich, St. Louis, MO, USA) was added and incubated for 10 min. The cells were then collected in RIPA buffer, electrophoresed, and immunoblotted using mouse anti-puromycin mAb clone 12D10 (1:1000; MilliporeSigma, Burlington, MA, USA). Membranes were incubated in secondary antibody HRP-conjugated anti-mouse IgG (1:10000; Cell Signaling Technologies, Danvers, MA, USA) and developed using Amersham ECL Prime Western Blotting Detection Reagent. ImageJ software from NIH was used to analyze the densitometry of immunoblots from three independent experiments.

### 2.10. Statistical Analysis

Differences between groups during infection were determined using two-way ANOVA and Bonferroni posttests. All results in the figures are shown as means ± SD from three to five independent experiments. The data were analyzed with GraphPad Prism version 8 software (GraphPad Software, La Jolla, CA, USA). 

## 3. Results

### 3.1. CHIKV Replicates more Efficiently in C8-D1A Cells than NSC-34 Cells

**Figure 1:**
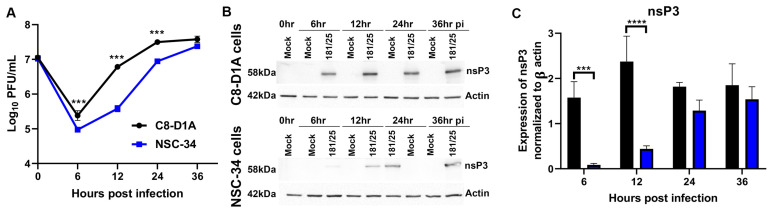
CHIKV replicates more efficiently in mouse C8-D1A astrocytic cells than NSC-34 neuronal cells. Cells were infected with CHIKV 181/25 at an MOI of 5 as measured by plaque assay on Vero cells. (**A**) Production of infectious virus by C8-D1A cells and NSC-34 cells. Data are presented as the geometric mean +/− SD from two independent experiments each performed in triplicate. *** *p* < 0.001 (**B**) Representative immunoblots for expression of nsP3 6, 12, 24 and 36 h post infection (hpi) of C8-D1A (top) and NSC-34 (bottom) cells. (**C**) Amounts of nsP3 produced in C8-D1A compared to NSC-34 cells after infection. Blots from three separate experiments were scanned, quantified with Image J, band densities normalized to actin, and ratios compared with Student’s *t* test. *** *p* < 0.001; **** *p* < 0.0001.

To directly compare the replication kinetics of CHIKV 181/25 (WT) in astrocytic and neuronal cell lines in vitro, well-characterized mouse C8-DIA astrocytic cells [60,64] and NSC-34 motor neuronal cells [53,61,65] were infected at an MOI of 5, and the production of infectious virus was quantified by plaque formation on Vero cells (Figure 1A). Virus was produced more rapidly and to higher titer throughout 24 h by C8-D1A cells compared to previously studied NSC-34 cells. A more rapid initiation of infection in C8-DIA cells than in NSC-34 cells was reflected in the earlier synthesis of nsPs evident in the expression of nsP3 at 6 h and 12 h after infection (Figure 1B,C).

### 3.2. nsP3 MD Function Affects the Initiation of CHIKV Infection in C8-D1A Cells

**Figure 2:**
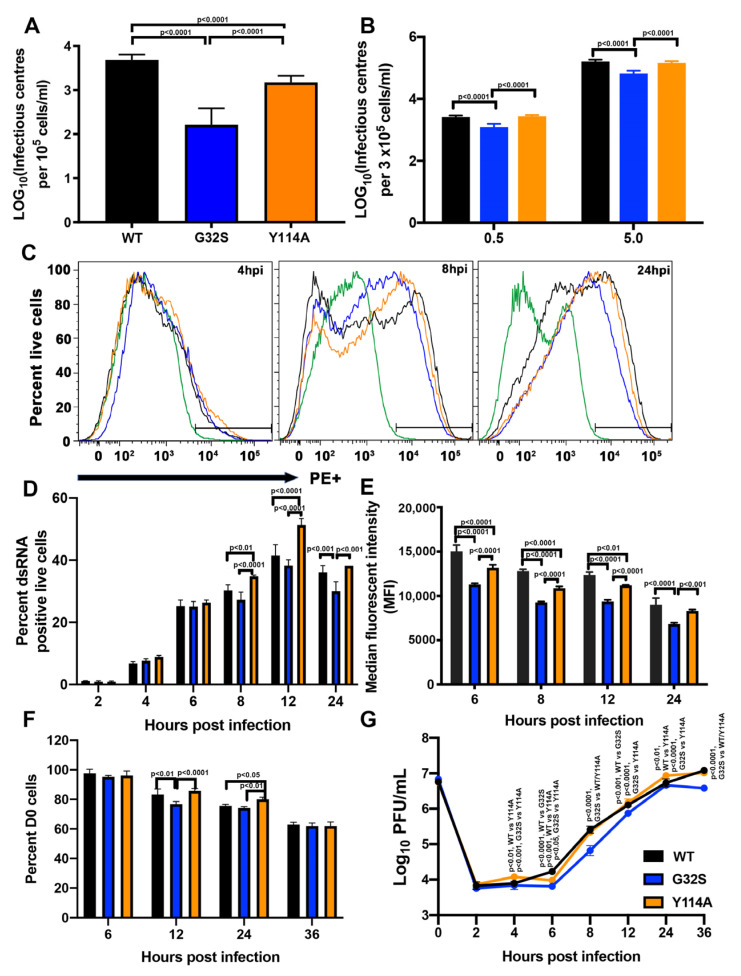
Effect of nsP3 MD mutations affecting ADPr-binding and hydrolase activities on initiation of infection and amplification of replication complexes in C8-D1A cells. (**A**) Infectious center assays for C8-D1A cells (10^5^ cells) electroporated with 10 µg of viral RNA of CHIKV 181/25 (WT) or nsp3 MD mutants G32S and Y114A. Electroporated cells were plated onto subconfluent BHK-21 cells and overlaid with bacto agar to identify virus-producing cells by plaque formation at 48 h. (**B**) Infectious center assays for C8-D1A cells infected with WT (black) or nsP3 MD mutants G32S (blue) and Y114A (orange) at MOIs of 0.5 and 5, incubated an hour at 4 °C and then 37 °C for 4 h. Cells were trypsinized, serially diluted, plated on Vero cells, and overlaid with bacto agar to assess plaque formation at 48 h. Data are presented as log_10_ infectious centers per 10^5^ cells and represent the mean ± SD from three independent experiments. (**C**) Representative histograms showing the formation and amplification of replication complexes in C8-D1A cells infected with WT (black) and nsP3 MD mutants G32S (blue) and Y114A (orange). Cells were infected at an MOI of 5, live/dead stained, fixed, permeabilized, and stained for dsRNA with J2 mAb and analyzed by flow cytometry. Green line indicates uninfected cells. (**D**) The percentages of live cells positive for dsRNA were quantified and compared at each time point and data presented as a bar graph. (**E**) Median fluorescent intensities presented as the mean ± SD from two independent experiments. (**F**) Cells infected with CHIKV WT, G32S and Y114A mutants at an MOI 5 were counted and cell viability was determined by trypan blue exclusion at 6, 12, 24 and 36 h after infection. (**G**) Supernatant fluids were collected, and infectious virus produced was determined by plaque formation. The data are presented as the mean ± SD from three independent experiments each performed in triplicate. *p* values indicate the significance of differences between WT and G32S/Y114A or between G32S and Y114A at the indicated time points.

To determine the roles of nsP3 MD ADPr binding and hydrolase activities for CHIKV replication in C8-D1A cells, WT infection was compared with infection by two recombinant CHIKVs with selective MD mutations previously characterized in and published for NSC-34 and BHK-21 cells [38,53]. G32S decreased ADP-ribosylhydrolase activity (~48% WT) and ADPr binding (~0.5 × WT) and Y114A decreased hydrolase activity (~41% WT) and increased ADPr binding (~4.7 × WT). To assess the initiation of infection in C8-D1A cells, infectious center assays were performed after electroporation of full-length RNAs transcribed from the cDNAs of WT and nsP3 MD mutant viruses to eliminate potential differences in virus attachment and entry (Figure 2A) and after infection with these viruses at low (0.5) and high (5) MOIs (Figure 2B). After electroporation or infection, C8-D1A cells were co-cultured with BHK-21 cells, overlaid with agar, and the number of plaques formed at 48h was counted to determine the numbers of C8-D1A cells in which infection was successfully established. For cells transfected with 10 μg RNA transcribed from full length cDNA clones of each virus, G32S RNA produced fewer infectious centers per 10^5^ cells (N = 163) than WT (N = 4831, *p <* 0.0001) and Y114A (N = 1491, *p* < 0.0001). Y114A also produced fewer infectious centers than WT (*p* < 0.0001) (Figure 2A). These differences were similar to those previously observed and published for NSC-34 cells and greater than for BHK-21 cells [53].

For virus infection, G32S also infected fewer C8-D1A cells than WT at both MOIs (1267 vs. 2592 per 3 × 10^5^ cells; MOI = 0.5, *p* < 0.0001); (67,667 vs. 162,500 per 3 × 10^5^ cells; MOI = 5, *p* < 0.0001). Y114A produced numbers of infectious centers that were similar to those produced by WT infection and more than G32S (1267 vs. 2742 per 3 × 10^5^ cells; MOI = 0.5, *p* < 0.0001); (67,667 vs. 145,000 per 3 × 10^5^ cells; MOI = 5, *p* < 0.0001) (Figure 2B). Therefore, nsP3 MD mutation decreases the likelihood of successful initiation of infection in C8-D1A cells as was previously published for NSC-34 cells [53].

### 3.3. nsP3 MD Function Affects Amplification of Replication Complexes and Virus Production by C8-D1A Cells

After replication complexes are established, they are amplified, increasing the numbers of spherules with dsRNA producing negative-strand template gRNA and positive-strand gRNA and sgRNA. To assess replication-complex amplification, C8-D1A cells infected with WT and G32S and Y114A mutants (MOI = 5) were stained with antibody to dsRNA and analyzed by flow cytometry (Figure 2C–E). At 4 and 6 hpi, cells infected with WT and mutants showed no difference in the numbers of dsRNA-positive cells (Figure 2D). However, at 8 and 12 hpi, more cells infected with Y114A were positive for dsRNA than cells infected with WT (*p* < 0.01 for 8 hpi; *p* < 0.0001 for 12 hpi) or G32S (*p* < 0.0001 for 8 and 12 hpi) (Figure 2C,D). Analysis of mean fluorescence intensity as a measure of amplification in individual cells indicated that WT-infected C8-D1A cells amplified more replication complexes than cells infected with G32S or Y114A at all times assessed (Figure 2E). Better ADPr-binding activity of Y114A compared to WT (4.84 μM vs. 22.9 μM K_D_) [38] favored viral infection despite a deficiency in hydrolase function. Therefore, in C8-D1A cells, as in NSC-34 cells, ADPr-binding activity was more important than hydrolase activity for establishing infection and amplifying replication complexes, supporting the likely importance of interaction with an ADP-ribosylated viral or host protein early in infection. 

To characterize the effect of nsP3 MD mutations on the entire CHIKV life cycle, we also assessed viability and infectious virus production by C8-D1A cells infected with WT, G32S, and Y114A viruses (MOI = 5). Cell viability was determined by trypan blue exclusion (Figure 2F) and infectious virus in supernatant fluids was quantified by plaque formation in Vero cells (Figure 2G). WT and Y114A produced viruses with similar kinetics and at 24 hpi, Y114A had more viable cells (*p* < 0.05) and higher viral titer (*p* < 0.01) than WT. The G32S virus replicated less well than WT and Y114A from 6 hpi (*p* < 0.0001 vs. WT, *p* < 0.05 vs. Y114A) until 36 hpi (*p* < 0.0001 vs. WT/Y114A). Cell numbers at 36 hpi were similar. 

### 3.4. nsP3 MD ADPr Binding Affects gRNA Translation and Viral RNA Synthesis in C8-D1A Cells

**Figure 3:**
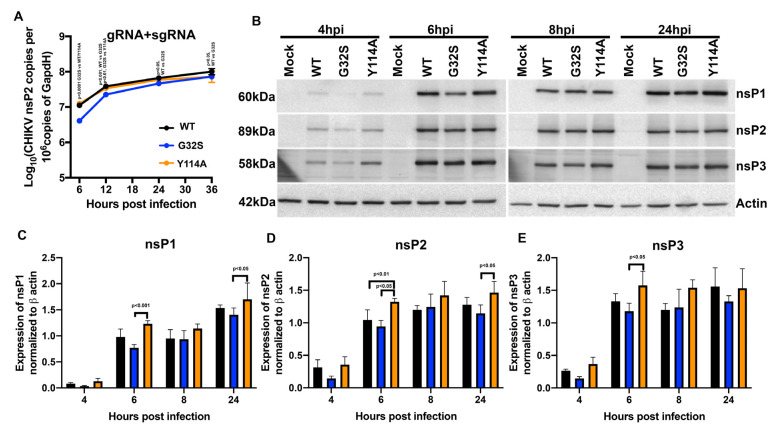
Effect of nsP3 MD function on astrocyte synthesis of viral RNAs and nsPs. C8-D1A cells were infected with CHIKV WT, G32S, and Y114A at an MOI of 5 and analyzed for synthesis of viral RNA and translation of nsPs. (**A**) Genomic and subgenomic (gRNA + sgRNA; E2) viral RNAs were quantified by qRT-PCR. The mean ± SD of log_10_ nsP2 or E2 copies/10^6^ copies of *Gapdh* from three independent experiments are plotted. (**B**) Representative immunoblots of viral nsP proteins at 4, 6, 8, and 24 h post infection (hpi). Lysates from C8-D1A cells infected with WT or nsP3 MD mutants were immunoblotted with antibodies to nsP1, nsP2, nsP3, and β-actin followed by secondary HRP-labeled anti-IgG were developed using a chemiluminescent detection reagent. Expression levels of nsP1 (**C**), nsP2 (**D**), nsP3 (**E**) normalized to actin were measured by densitometry from three independent experiments and the values plotted as bar graphs of the mean ± SD. *p* values indicate the significance of differences between WT and G32S/Y114A or between G32S and Y114A at the indicated time points.

In spherules containing replication complexes, gRNA and sgRNA are transcribed from the negative-sense gRNA template. To assess viral RNA synthesis in WT and nsP3 MD mutant-infected C8-D1A cells, sgRNAs plus gRNAs (E2 primers) were quantified by RT-qPCR (Figure 3A). Levels of G32S RNA were lower compared to WT and Y114A at 6 hpi (*p* < 0.001 vs. WT; *p* < 0.0001 vs. Y114A) but similar later in replication (Figure 3B).

To determine whether impaired G32S initiation of infection was associated with the decreased translation of gRNA to produce nsPs, immunoblots for nsP1, nsP2, and nsP3 were performed on lysates of cells at 4, 6, 8, and 24 hpi (Figure 3B). Relative amounts of each protein were determined by scanning the blots and normalizing values to β-actin (Figure 3C–E). In general, G32S nsP synthesis was slower and Y114A synthesis was faster than WT (6 hpi: G32S vs. Y114A, *p* < 0.001 nsP1, *p* < 0.05 nsP2 and nsP3; Y114A vs. WT *p* < 0.01 nsP2). Therefore, the increased ADPr-binding ability of Y114A was associated with the formation of more dsRNA-positive cells and more rapid production of nsPs.

### 3.5. Effect of nsP3 MD Function on Host Translational Shut-off in CHIKV-infected C8-D1A Cells

**Figure 4:**
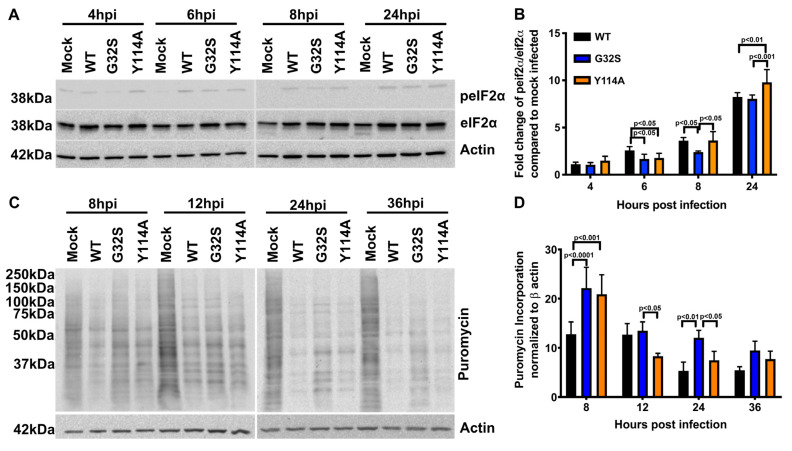
Effect of nsP3 MD function on host translational shut-off in CHIKV-infected astrocytes. C8-D1A cells were infected with CHIKV 181/25 (WT) or nsP3 MD mutants G32S and Y114A at an MOI of 5. (**A**) Representative immunoblots of lysates from cells 4, 6, 8, and 24 h post infection (hpi) probed for phospho-eIF2ɑ, total eIF2ɑ, and β actin. (**B**) Ratios of phospho-eIF2ɑ to total eIF2ɑ were determined by densitometry and normalized to β actin. Data from 3 experiments are plotted as the mean fold change relative to mock-infected C8-D1A cells. (**C**) Representative immunoblots of lysates from C8-D1A cells incubated at 8, 12, 24, and 36 h after infection with medium containing puromycin and probed with antibodies to puromycin and β actin. (**D**) Puromycin incorporation was normalized to β actin and data plotted as a graph. Change in puromycin incorporation is indicated as the average ± SD from three independent experiments. *p* values indicate the significance of differences between WT and G32S/Y114A or between G32S and Y114A at the indicated time points.

In vertebrate cells, alphavirus infection leads to the activation of PKR, phosphorylation of eIF2α, and shutoff of host-protein synthesis, events that accompany the switch from translation of gRNA to the translation of sgRNA. To determine the effect of nsP3 MD mutations on the initiation of this host response in C8-D1A cells, we evaluated the induction of eIF2α phosphorylation by immunoblotting cell lysates for phospho-eIF2α (p-eIF2α) and total eIF2α (Figure 4A,B). Phosphorylation of eIF2α was detected early (4 hpi) and occurred in all infected cells. At 6 hpi, the ratio of p-eIF2α to total eIF2α was higher in WT-infected C8-D1A cells than G32S and Y114A-infected cells (*p* < 0.05) and at 24 hpi Y114A-infected cells had a higher p-eIF2α to eIF2α ratio than WT (*p* < 0.01) or G32S (*p* < 0.001)-infected cells.

The shut-off of host-protein synthesis was evaluated by the incorporation of puromycin, a structural analog of aminoacyl tRNA, into newly synthesized proteins (Figure 4C,D). The puromycin translation assay showed less translational shut-off in G32S and Y114A-infected cells than WT-infected cells at 6 h and a continued delay in shut-off with G32S infection compared to WT.

### 3.6. Translation of Structural Proteins from sgRNA in C8-D1A and NSC-34 Cells Is Differentially Affected by Altered nsP3 MD Function

**Figure 5:**
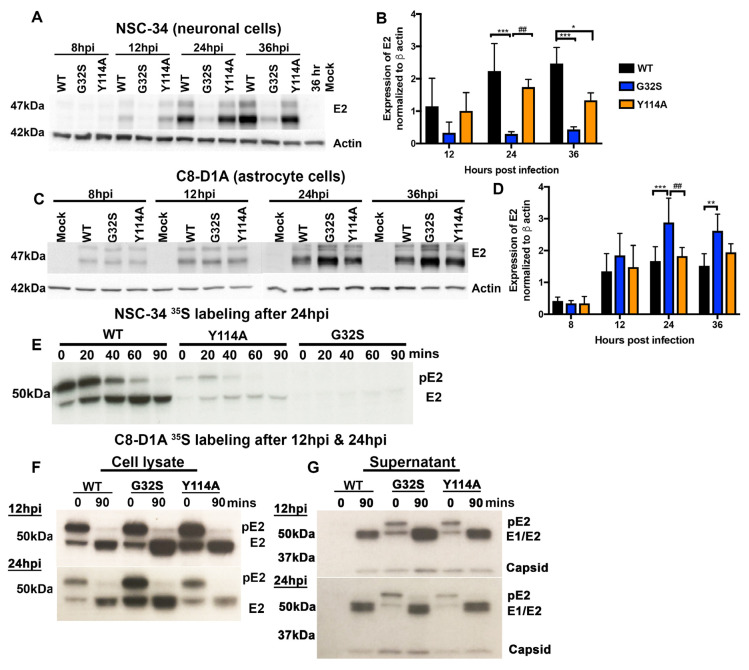
The G32S nsP3 MD mutation that affects both ADPr binding and hydrolase functions differentially affects structural protein synthesis by NSC-34 neuronal and C8-D1A astrocytic cells. NSC-34 and C8-D1A cells were infected with CHIKV WT, G32S, and Y114A at an MOI of 5. Representative immunoblots (**A**) and densitometry quantification (**B**) data on lysates from infected NSC-34 cells probed with antibodies to E2 glycoprotein and β-actin at 12, 24, and 36 h from Abraham et al., 2018 [53]. Representative immunoblots (**C**) and densitometry quantification (**D**) of lysates from infected C8-D1A cells probed with antibodies to the E2 glycoprotein and β-actin at 8, 12, 24, and 36 h. The averages from four independent experiments +/− SD are plotted as a bar graph. * *p* < 0.05; ** *p* < 0.01, *** *p* < 0.001 compared to WT; ^##^ *p* < 0.01 G32S compared to Y114A. For analysis of pE2 processing, cells were pulsed with ^35^S-methionine at 12 hpi and/or 24 hpi and chased for 90 min. Cell lysates were immunoprecipitated with antibody to E2 then subjected to polyacrylamide gel electrophoresis, fixed, dried, exposed to X-ray film, and developed. Representative images from pulse-chase analyses of infected NSC-34 (**E**) and C8-D1A (**F**) cells. Pulse-chase analysis of NSC-34 cells was performed once. The image for C8-D1A cells is representative of three independent experiments. (**G**) PEG-precipitated virions from 12 and 24 h pulse-chase C8-D1A cell supernatant fluids (panel E) were subjected to polyacrylamide gel electrophoresis, fixed, dried, exposed to X-ray film, and developed. Image is representative of three independent experiments.

In contrast to the translation of viral nsPs from gRNA, translation of structural proteins from sgRNA occurs after the shut-off of host-protein synthesis and is independent of translation-initiation factors eIF2(α/δ) and eIF4(e/f/g) [66]. To evaluate the effects of altered nsP3 MD ADPr-binding and hydrolase functions on structural protein synthesis, we performed immunoblot analyses of E2 protein expression by infected C8-D1A and NSC-34 cells (Figure 5). In NSC-34 neuronal cells, G32S synthesis of E2 was severely impaired compared to WT and Y114A, while E2 expression by Y114A-infected cells was at levels similar to WT (Figure 5A,B). In contrast, in C8-D1A astrocytic cells, G32S synthesis of E2 was greater than that of either WT or Y114A at 24 hpi (*p* < 0.001 vs. WT; *p* < 0.01 vs. Y114A) and greater than WT at 36 hpi (*p* < 0.01) while Y114A E2 synthesis was similar to WT (Figure 5C,D).

To determine whether nsP3 MD mutations affected the processing of precursor pE2 to mature E2, pulse-chase experiments were performed. NSC-34 and C8-D1A cells were pulsed with ^35^S-methionine for 30 min either 24 h after infection (Figure 5E) or 12 h and 24 h after infection (Figure 5F) and chased for 90 min. Lysates were immunoprecipitated with antibody to E2 and the labeled proteins examined by polyacrylamide gel electrophoresis (PAGE) and autoradiography. During the pulse, G32S synthesis of pE2 by NSC-34 cells was decreased compared to WT and Y114A (Figure 5E) and synthesis of pE2 by C8-D1A cells was increased (Figure 5F). However, the processing of pE2 to E2 was not affected by mutations in the nsP3 MD and occurred over a similar time course for all viruses and both cell types.

To determine whether the increased synthesis of structural proteins by C8-D1A cells infected with G32S was reflected in an increased release of viral particles, virus was precipitated from the pulse-chase supernatant fluids with polyethylene glycol at 12 and 24 h after infection and examined by PAGE and autoradiography (Figure 5G). At 12 hpi, more labeled virus particles were precipitated from G32S-infected C8-D1A cells than from Y114A- or WT-infected cells, but at 24 hpi the amounts were similar. Therefore, C8-D1A cells infected with nsP3 MD mutant G32S produced more structural proteins than cells infected with WT or Y114A viruses, and these proteins were processed, assembled, and released as virus particles.

### 3.7. G32S Virions Produced by C8-D1A Cells Are less Infectious than WT or Y114A Virions

**Figure 6:**
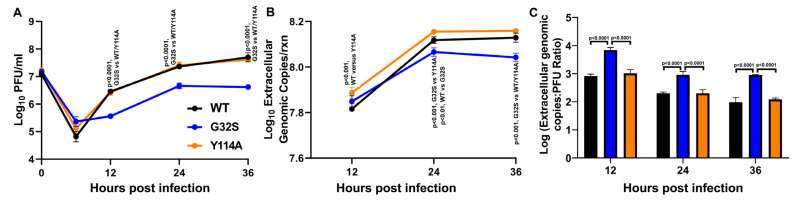
Infectivity of virions released from C8-D1A cells infected with CHIKV WT, G32S, and Y114A. C8-D1A cells were infected with CHIKV WT and nsP3 MD mutants G32S and Y114A at an MOI 5 measured by plaque assay on Vero cells. (**A**) Infectious extracellular virus released into the supernatant fluid as measured by plaque formation on Vero cells. (**B**) Genomic viral RNA (gRNA) released into supernatant fluid as quantified by qRT-PCR and displayed as log_10_ extracellular genome copies. (**C**) Ratios of genome copies: PFU for extracellular virions as calculated by converting qRT-PCR genomic copy per reaction data to genome copies per milliliter and dividing by PFU/mL at that time point. Values are an average of 3 biological replicates ± SD. *p* values indicate the significance of differences between G32S and WT or Y114A at each time point.

To assess the relative infectiousness of virions released from WT-, G32S-, and Y114A-infected C8-D1A cells, supernatant fluids were assayed for infectious virus by plaque assay (Figure 6A). gRNA was quantified (Figure 6B) and the ratio of gRNA copies to PFU was calculated at each time point (Figure 6C). As previously observed for Sindbis virus in BHK cells [67], particle infectivity improved as infection progressed for all the viruses, but virions produced by cells infected with the G32S mutant virus were less infectious than virions produced by Y114A- and WT-infected C8-D1A cells at all time points. These data are consistent with decreased infectious virus production by G32S-infected C8-D1A cells compared to WT- and Y114A-infected cells (Figure 2G).

## 4. Discussion

These studies have shown that more efficient replication of CHIKV in C8-D1A astrocytic cells than NSC-34 neuronal cells is associated with a more rapid translation of gRNA to produce the nsPs necessary for the initiation of replication-complex formation. As previously reported for NSC-34 cells, the initiation of infection was affected by mutations in the MD of nsP3 that alter binding to and removal of ADPr from ADP-ribosylated substrates. Greater binding (Y114A) was associated with more rapid synthesis of nsPs and more efficient initiation of infection despite decreased hydrolase activity. Mutations that decrease both ADPr-binding and hydrolase function (G32S) impair the initiation and amplification of infection, leading to reduced virus production by astrocytic as well as neuronal cells. However, the translation of sgRNA to produce viral structural proteins was differentially affected in NSC-34 cells and C8-D1A cells by the G32S MD mutation. Synthesis was decreased in NSC-34 cells and increased in C8-D1A cells, but the virus produced by G32S-infected C8-D1A cells was less infectious than that produced by WT and Y114A-infected cells. Therefore, nsP3 MD mutations that affect interactions with ADP-ribosylated host or viral proteins result in cell-type-dependent effects on CHIKV replication likely to be important for neurovirulence.

Many factors may contribute to the cell-type-dependent efficiency of virus replication and cell tropism. These include the availability of entry receptors and host factors required for virus replication, as well as the induction of innate antiviral responses. As for other alphaviruses, CHIKV replicates more efficiently in immature cells and is sensitive to the antiviral effects of IFN [68]. The proteomic analysis of CHIKV infection of the IFN-defective human astrocyte cell line U-87 MG showed major changes in proteins associated with transcription, translation, apoptosis, stress, and ubiquitylation [7]. Direct comparisons of the proteomes and transcriptomes, as well as the innate responses to infection, of astrocytes and neurons might provide insights into the differential support for or restriction of CHIKV replication in these two neural cells. Of particular interest would be host factors that facilitate successful infection after genome delivery.

Although CHIKV initiated infection of C8-D1A cells more rapidly than NSC-34 cells, the effect of the G32S and Y114A nsP3 MD mutations were similar in the two cell types. For both neuronal and astrocytic cells, the initiation of infection was facilitated by an increased affinity of the MD for ADPr [53]. Improved ADPr binding of mutant Y114A largely compensated for the hydrolase deficit in the initiation of infection while mutant G32S with decreased ADPr-binding and hydrolase activities initiated infection less efficiently than WT [53]. The HVD of nsP3 is important for cell-type-dependent recruitment of host factors into replication complexes [39,69] with the possibility that for ADP-ribosylated proteins, interactions are strengthened by binding to the MD. For instance, translation of CHIKV gRNA is facilitated by the interaction of the nsP3 HVD with DExH-box helicase 9 (DHX9), a protein that can be MARylated by PARPs 10 and 14 [70,71,72]. In addition, G3BP1, a highly regulated multifunctional RBP, is critical for establishing replication complexes and can be ADP-ribosylated. G3BP1 binds FGDF motifs in the HVD of nsP3 and interacts with gRNA and the 40S ribosomal subunits to facilitate the translation of the CHIKV nonstructural polyprotein [42,71,73,74,75]. Future studies to identify and examine the role of ADP-ribosylated proteins in replication complexes and their interactions with the nsP3 MD will be of interest.

In addition to a role in the initiation of CHIKV infection, G3BP also facilitates innate response signaling and stress-granule formation [76,77,78,79]. Stress granules form in response to the phosphorylation of eIF2α and the cessation of protein synthesis. With the cessation of protein synthesis, translating polysomes are disassembled and induce the formation of aggregates of stalled translation-initiation complexes, miRNAs, innate response factors, and RBPs, which are dependent on ADP-ribosylation [40,78,80,81]. To make otherwise sequestered host proteins available for viral protein synthesis, many viruses inhibit stress-granule formation or disrupt stress granules after induction [82,83,84,85,86,87,88]. The translation of sgRNA to produce alphavirus structural proteins is independent of eIF2α and occurs in the absence of host-protein synthesis [66]. Alphavirus nsP3 disrupts stress granules to form different cytoplasmic granules that contain G3BP and other RBPs, but not translation factors [41,89,90,91] and the ADP-ribosylhydrolase activity of the WT nsP3 MD is critical for stress-granule disruption [42]. 

We postulate that these translation factors become available for the translation of sgRNA while RBPs that regulate sgRNA translation [92,93] continue to be sequestered with nsP3 [38,42,53]. Current studies show that infection with CHIKV nsP3 MD mutant G32S that is deficient in both binding and hydrolase activity has a dramatically different effect on the translation of sgRNA in NSC-34 and C8-D1A cells, even though levels of sgRNA are similar. In NSC-34 cells, very little E2 protein was produced in comparison with WT, while in C8-D1A cells, more E2 protein was produced.

Because a deficiency in hydrolase activity delays nsP3-mediated disruption of stress granules and the release of translation-initiation factors [42], we postulate that in NSC-34 cells a decreased availability of translation factors is the primary determinant of decreased structural protein synthesis, while that is not the case in C8-D1A cells. The synthesis of structural proteins is regulated by the core RBPs hnRNPK, hnRNPI, and hnRNPM [92], members of a family of RBPs associated with RNA processing, trafficking, and translation. The mutation of the hnRNP binding sites on the sgRNA of Sindbis virus, a related alphavirus, to decrease hnRNP binding increases the translation of viral structural proteins, suggesting that the production of structural proteins needs to be curtailed for optimal virus production [93]. In addition to the RNA sequence, ADP-ribosylation of RBPs regulates binding to RNA so that less ADP-ribosylation increases RNA binding [94,95]. G3BP1 and hnRNPK are extensively ADP-ribosylated stress-granule proteins [40] that remain associated with nsP3 [92,93,96,97,98]. The hydrolase activity of WT nsP3 removes ADPr from G3BP1 and, we postulate, also from hnRNPK, which is not only ADP-ribosylated, but also binds ADPr [99] and is present in nsP3 granule complexes [42]. Therefore, a failure to remove ADPr from a regulatory RBP similar to hnRNPK, due to defective nsP3 MD ADPr hydrolase activity, will decrease binding to sgRNA, which in turn will increase structural protein translation and result in the release of more viral particles. 

The reason for the decreased infectivity of virus particles released from G32S-infected cells is not known, but this was also observed in previous studies of altered hnRNP binding to sgRNA with increased structural protein synthesis [100]. The infectivity of released virus particles generally improves as infection progresses, and this pattern was observed in our studies of viruses produced by C8-D1A cells. Because genomes must be capped to be infectious, it is possible that capping becomes more efficient later in infection with an accumulation of nsP1, and that overproduction of structural proteins results in virions being assembled with genomes that have not been capped or with defective viral genomes [101]. Alternatively, as identified for virions produced in response to increased structural protein synthesis associated with decreased hnRNPI binding to the 3′UTR of SINV sgRNA, glycosylation of the E1 and E2 glycoproteins may be defective [100]. In addition, the G32S phenotype is likely exaggerated by the impaired infectivity of the RNA associated with the loss of MD function.

Therefore, nsP3-dependent differences in alphavirus replication between NSC-34 and C8-D1A cells may reflect the cell-dependent availability of both translation factors and regulatory RBPs, their post-translational modifications, and changes induced by infection. Combined observations of these different cell types suggest that nsP3 MD hydrolase activity controls the availability of translation factors critical for sgRNA translation and structural protein synthesis and also regulates the ADP-ribosylation state of RBPs that program the level of sgRNA translation.

## Data Availability

Data not included in the manuscript will be available from the primary authors on request.
